# MiRNA-199a-3p Regulates C2C12 Myoblast Differentiation through IGF-1/AKT/mTOR Signal Pathway

**DOI:** 10.3390/ijms15010296

**Published:** 2013-12-27

**Authors:** Long Jia, Yue-Feng Li, Guo-Fang Wu, Zi-Yi Song, Hong-Zhao Lu, Cheng-Chuang Song, Qiang-Ling Zhang, Jia-Yu Zhu, Gong-She Yang, Xin-E Shi

**Affiliations:** Laboratory of Animal Fat Deposition and Muscle Development, College of Animal Science and Technology, Northwest A&F University, Yangling 712100, China; E-Mails: xinongjialong@163.com (L.J.); fengfeng0419@163.com (Y.-F.L.); letitbe521@163.com (G.-F.W.); songziyi89@gmail.com (Z.-Y.S.); zl780823@sina.com (H.-Z.L.); chengchuangsong@163.com (C.-C.S.); langyan.95@163.com (Q.-L.Z.); jiayuzhulam@163.com (J.-Y.Z.); gsyang999@hotmail.com (G.-S.Y.)

**Keywords:** miR-199a-3p, C2C12, muscle, myogenic differentiation

## Abstract

MicroRNAs constitute a class of ~22-nucleotide non-coding RNAs. They modulate gene expression by associating with the 3′ untranslated regions (3′ UTRs) of messenger RNAs (mRNAs). Although multiple miRNAs are known to be regulated during myoblast differentiation, their individual roles in muscle development are still not fully understood. In this study, we showed that miR-199a-3p was highly expressed in skeletal muscle and was induced during C2C12 myoblasts differentiation. We also identified and confirmed several genes of the IGF-1/AKT/mTOR signal pathway, including *IGF-1*, *mTOR*, and *RPS6KA6*, as important cellular targets of miR-199a-3p in myoblasts. Overexpression of miR-199a-3p partially blocked C2C12 myoblast differentiation and the activation of AKT/mTOR signal pathway, while interference of miR-199a-3p by antisense oligonucleotides promoted C2C12 differentiation and myotube hypertrophy. Thus, our studies have established miR-199a-3p as a potential regulator of myogenesis through the suppression of IGF-1/AKT/mTOR signal pathway.

## 1. Introduction

The genesis of skeletal muscles is a multi-step process that includes the recruitment of myoblasts from myogenic precursors, myoblast proliferation, cell cycle arrest and fusion of myocytes into multinucleated myotubes [1]. The highly complicated process of myogenesis is orchestrated by the muscle specific regulatory transcription factors (MRFs), including myogenic differentiation antigen (MyoD), myogenin (MyoG), myogenic factor 5 (Myf5), and myogenic regulatory factor 4 (MRF4). Myf5 and MyoD are mainly involved in controlling myoblast proliferation and early differentiation, while MyoG and MRF4 are intermediate and later markers of myogenic differentiation and required for myotube formation [2].

Recently, microRNAs, a class of evolutionarily conserved and small non-coding RNAs [3], have emerged as novel and essential regulators of myogenesis [4]. They vary from 17 to 24 nucleotides in length and can induce mRNA degradation or translation inhibition by interacting with the 3′ UTRs of their target mRNAs [3]. Muscle-specific miRNAs have a central role in myogenesis, such as miR-1 [5], miR-133 [6], and miR-206 [6]. Several ubiquitously expressed miRNAs have also been found to participate in myogenesis, including miR-26a [7], miR-27b [8], miR-29 [9], miR-125b [10], miR-155 [11], miR-181 [12], and miR-214 [13,14]. Although increasing number of miRNAs are found to function in myognenesis, knowledge about individual roles of miRNAs in muscle development remains limited.

There are two loci within the human genome that encode the precursor of miR-199a-3p: one is embedded in the antisense chain of intron 15 of Dynamin 2, referred to as miR-199a-1; the other is in the antisense chain of intron 14 of Dynamin 3, named miR-199a-2 [15]. Dynamin 2 is required for muscle development and its mutation causes centronuclear myopathy in skeletal muscle [16]. The dynamin 3 locus encodes an antisense transcript, Dnm3os, which gives rise to miR-199a-2 and another microRNA, miR-214. Dnm3os is required for normal skeletal development in mice and its deficiency also influences muscle development [17]. MiR-214 has been demonstrated to participate in myogenic differentiation by facilitating myoblasts exit from mitosis [14]. However, little is known about the role of miR-199a in muscle development.

In this study, we shown that miR-199a-3p is an extensively expressed miRNA and highly expressed in skeletal muscle, especially in soleus and induced during C2C12 myoblast differentiation. Using bioinformatic analyses and luciferase reporter assay, we found that miR-199a-3p acts to target the 3′ UTR of several genes in the IGF-1/AKT/mTOR signal pathway, including *IGF-1*, *mTOR*, and *RPS6KA6*. Over-expression and interference of miR-199a-3p in C2C12 cells resulted in decreased and increased expression of IGF-1, mTOR and RPS6KA6. Transfection of miR-199a-3p mimics inhibited differentiation of C2C12 myoblasts, resulting in decreased proportion of MyHC^+^ cells and expression of myogenic marker genes, *MyoD*, *Myf5*, *MyoG* and *MyHC*. MiR-199a-3p interference by antisense oligonucleotides promoted C2C12 myoblast differentiation and myotube hypertrophy. Taken together, these studies demonstrate that miR-199a-3p is a potential regulator of myogenesis that acts to suppress the IGF-1/AKT/mTOR signal pathway.

## 2. Results

### 2.1. Tissue Expression Profile of MiR-199a-3p and Its Expression Pattern during C2C12 Myogenic Differentiation

**Figure 1.**
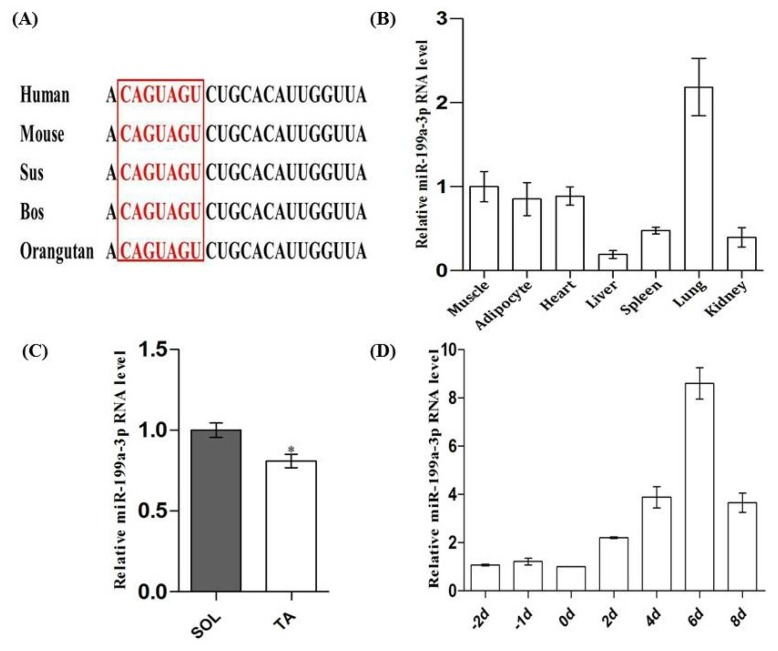
Tissue expression profile of miR-199a-3p and its expression pattern during C2C12 myogenic differentiation. (**A**) miR-199a-3p is conserved in mammals; (**B**) Tissue distribution of miR-199a-3p in adult mice; (**C**) Expression of miR-199a-3p in SOL (soleus: slow muscle) and TA (tibialis anterior: fast muscle) of adult mice; and (**D**) The expression of miR-199a-3p during C2C12 myoblast differentiation. Results are presented as mean ± S.E.M. *n* = 3. * *p* < 0.05.

MiR-199a-3p is conserved in mammals (Figure 1A). To determine the tissue expression profile of miR-199a-3p, total RNAs from seven tissue types were collected from adult Kuming mice (Fourth Military Medical University, Xi’an, China) and real-time qPCR was performed. Results showed that miR-199a-3p was highly expressed in skeletal muscle and lung (Figure 1B), which was consistent with previously published findings [18]. We then detected the expression of miR-199a-3p in different types of muscles. We founded that miR-199a-3p displays slightly higher expression levels in the slow myofiber enriched soleus muscle compared to the tibialis anterior muscle that is enriched in fast myofibers (Figure 1C). Previously, Chen *et al.* discovered that the expression of miR-199a-3p was increased during C2C12 myoblast differentiation by gene microarray [5]. We used real-time qPCR to confirm this. The results showed that miR-199a-3p has an increased expression pattern during myogenic differentiation of C2C12 cell lines (Figure 1D).

### 2.2. Analysis of MiR-199a-3p Target Genes

**Figure 2.**
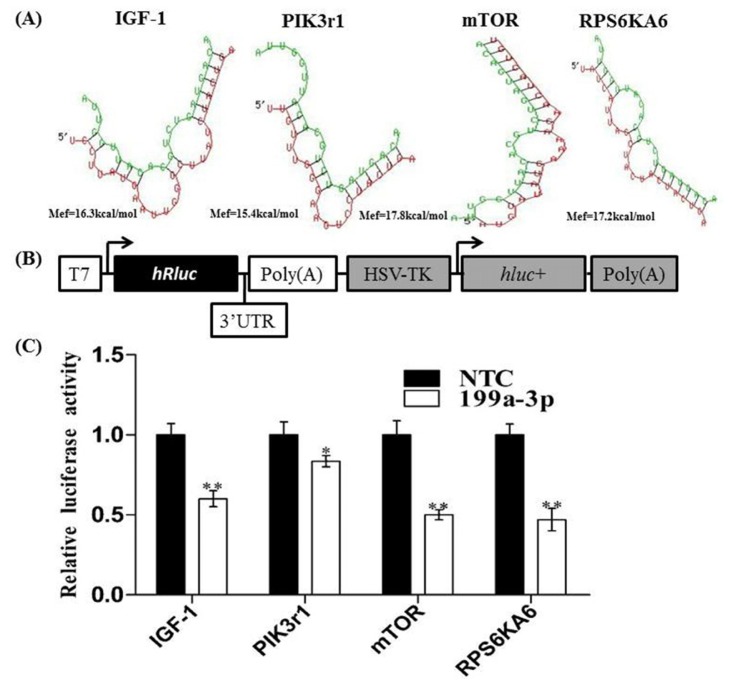
MiR-199a-3p targeted several components of the IGF-1/AKT/mTOR signaling pathway. (**A**) The schematic of miR-199a-3p binding to its targeted genes using RNAhybrid, the green sequence is miR-199a-3p mature sequence, red is the sequence of miR-199a-3p targeted gene; (**B**) The schematic of the psiCHECK™-2 luciferase reporter vector; and (**C**) The 3′ UTR luciferase reporter vectors of mouse IGF-1, PIK3R1, mTOR, RPS6KA6 containing miR-199a-3p targeting sites, were co-transfected with miR-199a-3p mimics (or negative control miRNA) into 293T cells. Thirty-six hours after transfection, luciferase assay was performed. The results are represented as mean ± S.E.M. *n* = 3. *p < 0.05, ** *p* < 0.01.

Using Target Scan 6.2 (http://www.targetscan.org/) and RNAhybrid, we found that miR-199a-3p may target several signal molecules involved in the IGF/AKT/mTOR signal pathway, including insulin-like growth factors (IGF-1), phosphatidylinositol 3-kinases regulatory 1 (Pik3r1; also referred to as p85a), the mammalian target of rapamycin (mTOR) and ribosomal protein S6 kinase, 90 kDa, polypeptide 6 (RKS6KA6) (Figure 2A).

To verify whether *IGF-1*, *PIK3r1 mTOR and RKS6KA6* were the target genes of miR-199a-3p, the 3′ UTRs of mouse IGF-1, PIK3r1 mTOR and RKS6KA6 containing miR-199a-3p targeted sites were amplified and cloned to psiCHECK™-2 Vector (Figure 2B). Then the luciferase reporter vectors containing the 3′ UTRs of these four genes were co-transfected into 293T cells along with miR-199a-3p mimic or negative control (NTC), respectively. Results showed that miR-199a-3p over-expression dramatically reduced the activity of reporters containing the 3′ UTRs of IGF, PIK3r1, mTOR, and RKS6KA6 (Figure 2C).

### 2.3. Over-Expression of MiR-199a-3p Inhibits C2C12 Myogenic Differentiation

**Figure 3.**
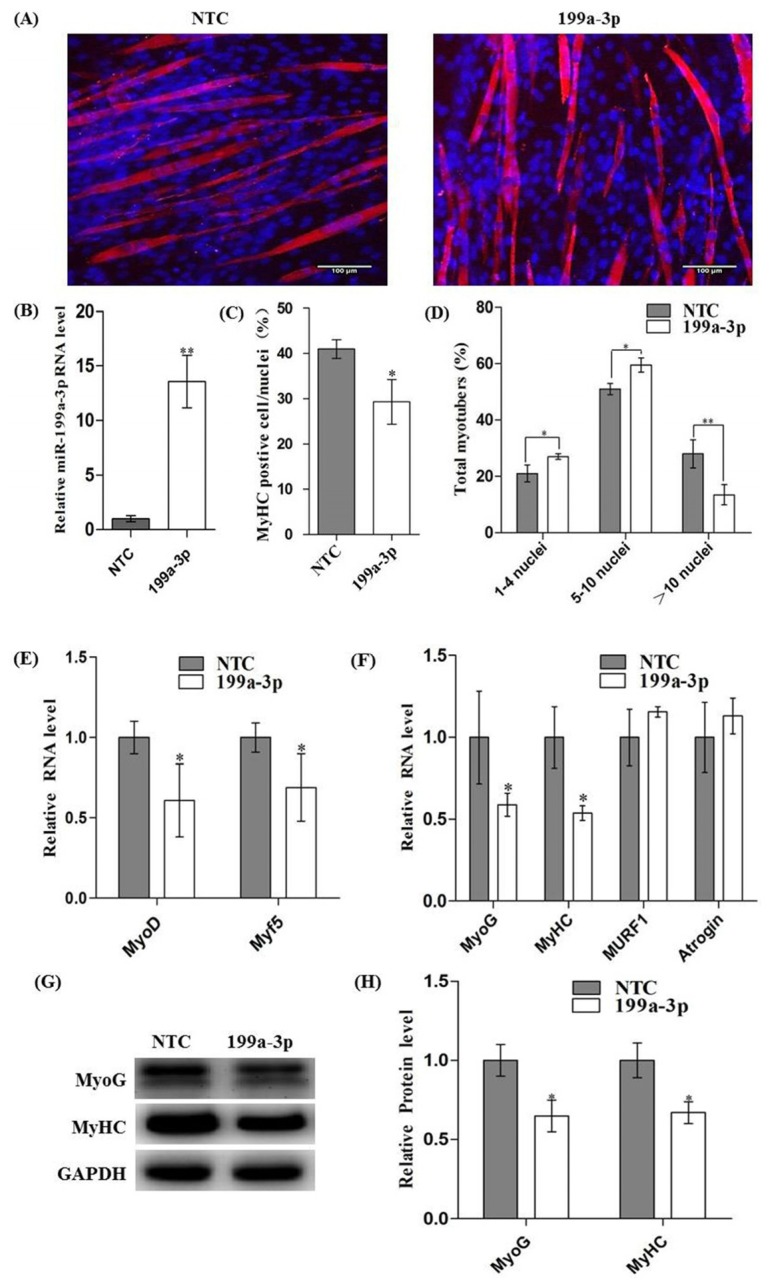
Overexpression of miR-199a-3p inhibited C2C12 myogenic differentiation. C2C12 cells were transfected with miR-199a-3p Mimics (or negative control miRNA) and grown in growth medium (GM) for 12 h, then induced to myogenic differentiation with differentiation medium (DM). At the 96 h of differentiation: (**A**) Immunofluorescence of muscle myosin heavy chain (MyHC) in C2C12 myotubes; (**B**) The over-expression efficiency was measured by real-time qPCR; (**C**) The fusion index was counted by MyHC-positive cells to total nuclei (Hoechest-positive cells); (**D**) Myotubes sizes were determined by myonuclei counts in myotubes transfected with miR-199a-3p mimic or negative control miRNA; (**E**) Relative mRNA levels of MyoD and Myf5 were detected by real-time PCR at the 24 h of differentiation; (**F**) Relative mRNA levels of MyoG, MyHC, MURF1 and Atrogin1 at the 96 h of differentiation; (**G**) Western Blot detected the protein expression of myogenic markers MyoG, and MyHC at the 96 h of differentiation; and (**H**) The relative protein levels in (**G**) were analyses using image J. Results are presented as mean ± S.E.M. *n* = 3. * *p* < 0.05, ** *p* < 0.01.

To establish the role of miR-199-3p in myogenic differentiation, C2C12 myoblasts were transfected with synthetic miR-199a-3p mimics or negative control (NTC). After 12 h, cells were induced differentiation with differentiation medium. Real-time qPCR showed that miR-199a-3p was successfully over-expressed (Figure 3B). The mRNA levels of MyoD and Myf5, early markers of myogenic differentiation, were decreased about 40% and 30% after 24 h of differentiation (Figure 3E). Ninety-six hours later, immunofluorescence of MyHC was performed to assess myotube formation. As showed in Figure 3A, myotubes were significantly decreased in cells expressing the miR-199a-3p mimic compared to the control, accompanied with a decreased proportion of MyHC^+^ cells (Figure 3C) and myotube sizes (Figure 3D). At the same time, both mRNA and protein levels of MyoG and MyHC, the intermediate and late markers of myogenic differentiation, were significantly decreased in cells transfected with miR-199a-3p mimics compared to the cells transfected with NTC (Figure 3F–H). The muscular atrophy related genes, *MURF1* and *Atrogin1* were slightly increased, but without significance (Figure 3F). Together, these results showed that over-expression of miR-199a-3p inhibited myoblast differentiation.

### 2.4. Inhibition of MiR-199a-3p Promotes Myogenic Differentiation and Myotube Hypertrophy

**Figure 4.**
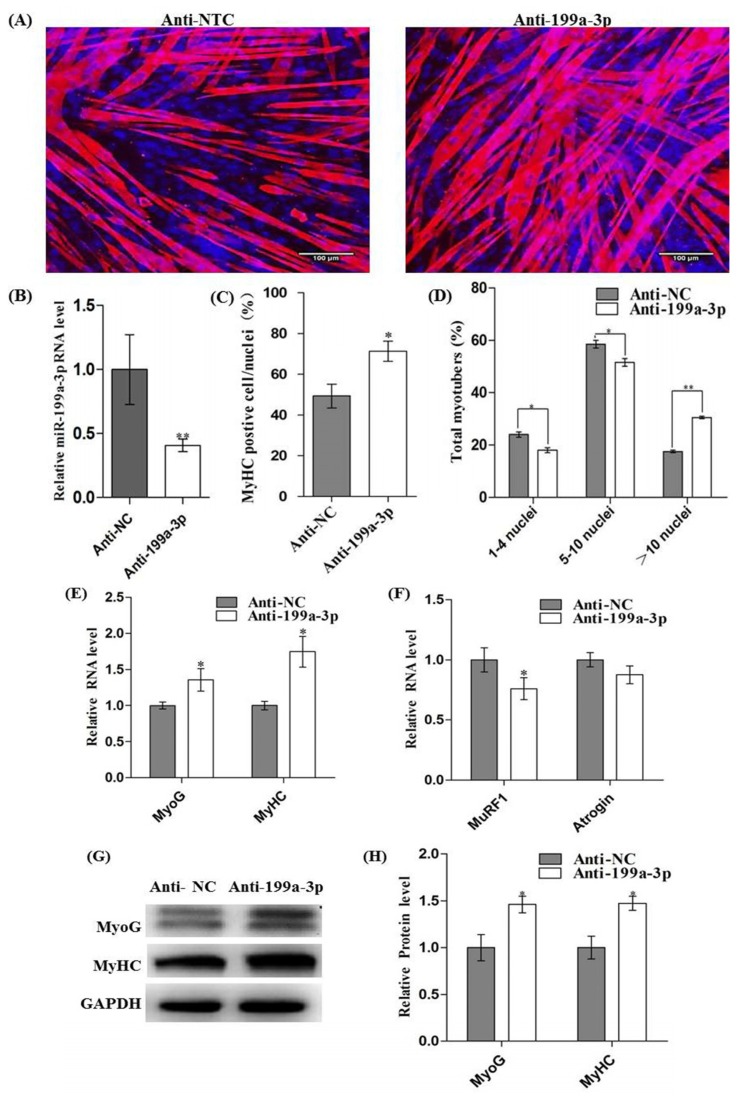
Inhibition of miR-199a-3p promoted myogenic differentiation. C2C12 cells were transfected with miR-199a-3p inhibitors (or negative control) and grown in GM for 12 h, then induced for myogenic differentiation for 96 h in DM. (**A**) Immunofluorescence of muscle myosin heavy chain (MyHC) in C2C12 myotubes; (**B**) The interference efficiency was measured by real-time qPCR; (**C**) Fusion index and (**D**) Myotubes sizes were analysed as described in Figure 3; (**E**) and (**F**) Relative mRNA levels of MyoG, MyHC, MURF1, and Atrogin were measured by real-time qPCR; (**G**) and (**H**) MyoG and MyHC protein levels were detected and quantitated as described in Figure 3. Results are presented as mean ± S.E.M. *n* = 3. * *p* < 0.05, ** *p* < 0.01.

To further demonstrate the role of miR-199a-3p in myogenic differentiation, the anti-sense oligonucleotides targeting miR-199a-3p or NC were transfected to C2C12 cells. After 12 h, cells were induced to differentiate. Immunofluorescence analysis (Figure 4A) showed that MyHC^+^ cells (Figure 4C) and myotubes sizes (Figure 4D) were markedly increased by miR-199a-3p inhibitor treatment at 96 h of differentiation. Real-time qPCR showed that miR-199a-3p was successfully interfered (Figure 4B) and the mRNA level of MyoG and MyHC were significantly upregulated in cells transfected with miR-199a-3p inhibitors (Figure 4E). Immunoblotting of MyoG and MyHC confirmed that they were also enhanced in protein level (Figure 4G,H). The muscular atrophy related genes, *MURF1* and *Atrogin1* were decreased along with miR-199a-3p inhibition (Figure 4F). These results demonstrated that interference of miR-199a-3p promotes myogenic differentiation and myotube hypertrophy.

### 2.5. MiR-199a-3p Affects IGF/AKT/mTOR Pathway in C2C12 Cells

**Figure 5.**
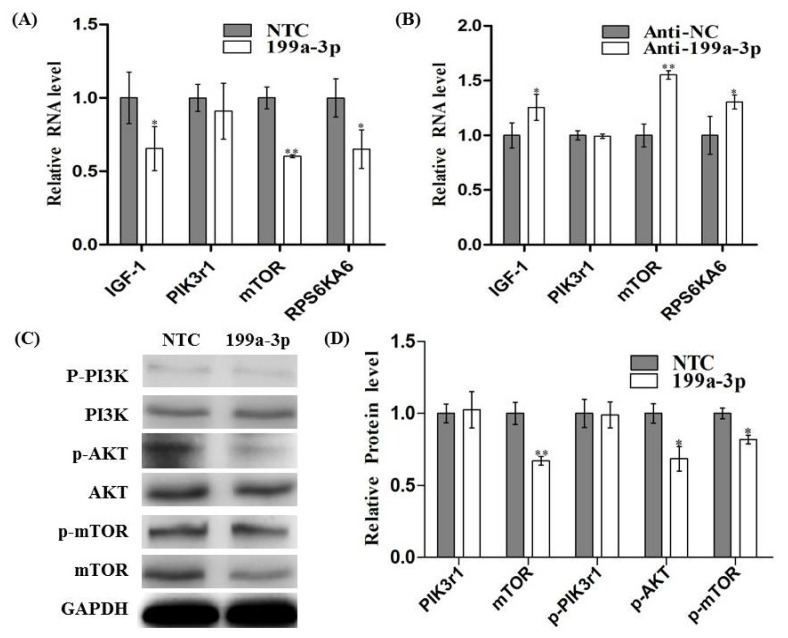
MiR-199a-3p regulated the IGF/AKT/mTOR pathway in C2C12 cells. (**A**) The mRNA expression of miR-199a-3p targeted genes, IGF-1, PIK3r1, mTOR, RPS6KA6, were detected by real-time PCR in C2C12 cells after transfection of miR-199a-3p mimic or negative control miRNA; (**B**) Real-time PCR detected IGF-1, PIK3r1, mTOR and RPS6KA6 mRNA expression after transfection of miR-199a-3p inhibitor or negative control into C2C12 cells; (**C**) Several signal molecules of IGF/AKT/mTOR signal pathway, including PIK3r1 (p85), p-PIK3r1 (p-p85), AKT, p-AKT, mTOR, p-mTOR were detected by Western Blot after transfection of miR-199a-3p mimic or negative control miRNA; and (**D**) protein expression levels were quantitated using image J. Results are presented as mean ± S.E.M. *n* = 3. * *p* < 0.05. ** *p* < 0.01.

In order to validate whether miR-199a-3p is a regulator of the IGF-1/AKT/mTOR signal pathway in myoblasts, we measured the mRNA and protein level of IGF-1, PI3Kr1, mTOR and RPS6KA6 after the transfection of miR-199a-3p mimic or inhibitor into C2C12 lines. We found that over-expression of miR-199a-3p leads to noteworthy decreased mRNA levels of IGF-1, mTOR, and RPS6KA6 (Figure 5A). When miR-199a-3p was knocked-down, the mRNA levels of IGF-1 mTOR and RPS6KA6 level were significantly increased (Figure 5B). However, we did not observe any changes in PIK3r1 mRNA and protein levels upon miRNA-199a-3p over-expression or inhibition. The decrease in AKT/mTOR pathway activity was confirmed through decreased phosphorylation levels of AKT and mTOR when miR-199a-3p was over-expressed (Figure 5C,D). These results suggest that miR-199a-3p may modulate the IGF-1/AKT/mTOR pathway to inhibit C2C12 myogenic differentiation.

## 3. Discussion

MiR-199a-3p is a conserved miRNA in mammals. A recent study showed that miR-199a-3p expression level is markedly elevated in muscle afflicted with Duchenne muscular dystrophy [19]. Similarly, TGF-β, a main inhibitor of skeletal muscle differentiation, can strongly induce the expression of miR-199a-3p [20]. However, whether miR-199a-3p has a role in myogenesis has not yet been addressed. Our results discussed herein showed that miR-199a-3p is widely expressed in multiple tissues of mammals including skeletal muscles and negatively regulates muscle development through IGF-1/AKT/mTOR pathway suppression.

The IGF-1/AKT/mTOR pathway lies at the heart of the regulatory network controlling muscle development [21]. Upon binding to its membrane receptor IGFR1, IGF-1 activates both ERK1/2 and PI3K/Akt/mTOR pathways. ERK1/2 is involved in myoblast proliferation [22], whereas the PI3K/Akt/mTOR pathway facilitates protein synthesis and is pivotal for myotube formation and muscle hypertrophy [23]. Several miRNAs have been demonstrated to regulate IGF-1/PI3K/AKT/mTOR pathway during myogenesis. MiR-1 [24] and miR-125b [10] counteract muscle hypertrophy by targeting IGF-1 and IGF-2 respectively. MiR-128a, also an anti-myogenic miRNA, can direct targeting to IRS1 and PIK3r1, critical mediators for activating the PI3K/AKT/mTOR pathway [25]. The muscle-enriched miR-486, however, is a well-established facilitator of IGF-1/Akt pathway and muscle development, which targets PTEN and Foxo1a, two inhibitors of IGF-1/Akt pathway [26].

Valeria *et al.* found that miR-199a-3p is up-regulated in the pancreas islet of adult diabetic mice [27]. Diwakar discovered that genes involved in PI3K/AKT pathway were down-regulated in NIH-3T3 cells when transfected with miR-199a-3p mimics [28]. Fornari *et al.* showed that miR-199a-3p can target mTOR in cancer cells. These facts suggest that miR-199a-3p may be a key regulator of the IGF-1/AKT/mTOR signaling pathway and here we confirm that miR-199a-3p targets several members of this pathway, including IGF-1, RKS6KA6, and mTOR. Over-expression of miR-199a-3p also induced a decrease in AKT phosphorylation levels, while we did not observe any change in both mRNA and protein level of PIK3r1. Recently, small RNAi-based gene silencing experiments revealed that IRS can directly phosphorylate Akt in skeletal muscle [29]. So, decreased phosphorylation of AKT may be due to the decreased IGF-1 level caused by miR-199a-3p over-expression.

Previous knowledge about the roles of miRNAs in myogenesis was obtained from muscle-specific miRNAs (Myo-miRs): miR-1, miR-133and miR-206, which target histone deacetylase 4 (HDAC4), serum response factor (SRF), and Follistatin-like 1 (FSTL1), respectively [6,7]. Shortly thereafter, some ubiquitously expressed miRNAs like miR-29 and miR-214 were also found to play important roles in myogenesis [9,13]. Studies in the past were largely focused on miRNAs regulating a single gene in myogenic signaling pathways. However, mounting evidence suggests that miRNA can also affect signal transduction pathways. MiR-128a exerts inhibitory effects on myogenesis by repressing both IRS1 and PIK3r1 of the insulin signaling pathway [25]. By targeting multiple members of the same signaling pathway, microRNA can exert more profound effects than regulating one individual gene in a biological process.

## 4. Materials and Methods

### 4.1. Cell Culture

C2C12 myoblasts (ATCC, Rockefeller, New York, NY, USA) were cultured in growth medium (GM, Dulbecco’s Modified Eagle Medium containing 10% fetal calf serum) before induced to differentiation, at 37 °C, 5% CO_2_. When cell density reached 70%, they were digested with 0.25% trypsin, and then seeded into culture dishes. When inducing C2C12 myoblasts to differentiate, cell density must reach >90% prior to changing GM to differentiation medium (DM, Dulbecco’s Modified Eagle Medium supplemented with 2% horse serum).

### 4.2. Transfection of MiR-199a-3p Mimic and Inhibitor

MiR-199a-3p mimic was double-stranded, whereas the inhibitor single-stranded. They were both designed and synthetized by GenePharama (Shanghai, China). MiR-199a-3p mimics Sense: 5′-ACAGUAGUCUGCACAUUGGUUA-3′; Antisense: 5′-ACCAAUGUGCAGACUACUGUUU-3′. miR-199a-3p inhibitor: 5′-UAACCAAUGUGCAGACUACUGU-3′. Negative controls of miR-199a-3p mimic or inhibitor were also purchased from GenePharama (Supplementary Table S1).

For transfection, cell density was allowed to reach 70%. C2C12 myoblasts were subjected to serum starvation for 4 h prior to transfection. MiR-199a-3p mimic or inhibitor were transfected into C2C12 using Lipofectamine2000 (Invitrogen, Carlsbad, CA, USA).

### 4.3. Immunocytochemical Analysis

After transfection and induced myogenic differentiation, C2C12 myoblasts cultured in 12-well plates were washed with PBS and fixed with 4% paraformaldehyde for 15 min. 0.5% Trixon-100 was used for permeabilization. The cells were then blocked in 2% goat serum (diluted in PBS). After blocking, the cells were incubated with the anti-myosin primary antibody at 37 °C for 1–2 h, and then the fluorescent secondary antibodies at 37 °C for 1 h. The nuclei were stained with Hoechest (Beyotime, Shanghai, China) for 10min. Images were captured using a Nikon TE2000 microscope (Nikon, Tokyo, Japan).

### 4.4. Real-Time qPCR

Trizol reagent (TaKaRa, Otsu, Japan) was utilized to extract tissue or cellular total RNAs. The total RNA quality and concentration were estimated using denatured gel electrophoresis and spectrophotometer (Thermo, Waltham, MA, USA), respectively. About 500 ng of the total RNA was processed into single strand cDNA using a reverse transcription kit (TaKaRa) with random primers. Real-time quantitative PCR was performed in triplicate using a SYBR green kit (TaKaRa) on a Bio-Rad iQTM5 system (Bio-Rad, Hercules, CA, USA). MicroRNA expression was normalized against the expression of U6 whereas GAPDH was used to normalize the expression of individual mRNAs. The 2^−ΔΔ^*^Ct^* algorithm was employed to estimate the relative expression level of each gene. The sequences of primers can be found in Supplementary Tables S2 and S3.

### 4.5. Western Blot Detection

Cell cultures of C2C12 were homogenized in ice-cold protein lysis buffer (RIPA, Beyotime, Shanghai, China) containing protease inhibitor (Pierce, Rockford, IL, USA). The lysates were centrifuged and the protein concentration of supernatant was determined using the Bicinchoninic Acid assay kit (Beyotime, Shanghai, China). Then 5× protein loading buffer was added to the lysates prior to full denaturation in boiled water. A total of 20 μg of protein was electrophoresed on a 12% SDS-polyacrylamide gel and transferred to a PVDF membranes (polyvinylidene difluoride membrane) (CST, Boston, MA, USA). The membrane was blocked in 5% defatted milk, and then incubated at 4 °C overnight with primary antibodies. Next day, the PVDF membranes were washed with TBST (tris-buffered-saline with tween) and incubated with second antibody at room temperature for 1 h and washed three times with TBST, and exposed and quantified using the Image J program (A software used for Luminance analysis, National Institutes of Health, Bethesda, MD, USA).

MyoD, MyoG, and MyHC antibodies were purchased from Developmental Studies Hybridoma Bank (University of Iowa, Iowa City, IA, USA); mTOR, phospho-mTOR, PI3K, phospho-PI3K, AKT, and phospho-AKT antibodies were from Santa Cruz Biotechnology (Dallas, TX, USA).

### 4.6. Luciferase Reporter Assay

The 3′ UTRs of mouse IGF-1, PI3Kr1, mTOR and RPS6kA6 genes containing miR-199a-3p targeted sites, were amplified by PCR using primers with the XhoI and Not I sites. The primers used were listed in Supplementary Table S4. The sequences were excised with XhoI and Not I and cloned into the psiCHECK™-2 Vector (Promega, Madison, WI, USA) at the 3′-end of the *Renilla* gene. The miR-199a-3p was co-transfected into 293T cells (ATCC) with the 3′ UTR dual-luciferase vectors using Lipofectamine 2000 (Invitrogen) and the medium replaced 6 h later. Cells were incubated for 36 h, and assayed according to the manufacturer’s instructions.

### 4.7. Statistic Analysis

Each experiment was repeated three times, all quantitative results are represented as mean ± S.E.M. One-way ANOVA in SPSS11.5 software (SPSS Inc., Chicago, IL, USA) was used to perform variance analysis and significance test, least significant difference (LSD) to tests the differences of different treatments.

## 5. Conclusions

In summary, our data identifies miR-199a-3p as a potential regulator of myogenesis through the suppression of the IGF-1/AKT/mTOR signal pathway. Recently, miR-199a-3p has been demonstrated to act as a direct regulator of COX-2 expression in human chondrocytes [30]. COX-2, an activator of AKT, is essential during early stages of skeletal muscle regeneration. Whether miR-199a-3p can also regulate COX-2 in skeletal muscle is yet to be unraveled. Several other bioinformatic predicted targets of miR-199a-3p, such as breast cancer anti-estrogen resistance 3 (BCAR3), cell cycle associated protein 1 (CAPRIN1), cell division cycle 42 (Cdc42), Yes-associated protein 1 (Yap1), and platelet-derived growth factor receptor, alpha polypeptide (PDGFRA) can also activate AKT. Together with our results, it is intriguing to speculate that miR-199a-3p might be a crucial regulator of the AKT/mTOR pathway and further investigations remain necessary to better establish its role in myogenesis.

## Supplementary Information
