# Supplementary material for: MiRNA-199a-3p Regulates C2C12 Myoblast Differentiation through IGF-1/AKT/mTOR Signal Pathway
Source: Int J Mol Sci. 2013 Dec 27;15(1):296–308. doi: 10.3390/ijms15010296 (PMC3907811; doi:10.3390/ijms15010296)
Supplement: Supplementary file 1 [file ijms-15-00296-s001.pdf]

Supplementary Information

Table S1. The Sequence of Negative Control for miRNA mimic and inhibitor.

Name	Sequence
NC(5' to 3')<br>(Control for mimics)	UUC UUC GAA CGU GUC ACG UTT<br>ACG UGA CAC GUU CGG AGA ATT
NC(5' to 3')<br>(Control for inhibitor)	CAG UAC UUU UGU GUA GUA CAA

Table S2. Primer sequences and parameters of Myoblast differentiation marker gene for real-time PCR.

Gene	Primer sequence (5' to 3')	Accession No.	T_m/°C
Myf5	F: GGACCAGTTTGAGCCAAGAG<br>R: CGAAAGCTTGGTTGACCTTC	NM_008656	55
MyoD	F: AGTGAATGAGGCCTTCGAGA<br>R: GCATCTGAGTCGCCACTGTA	NM_010866	60
MyoG	F: CAATGCACTGGAGTTCGGT<br>R: CTGGGAAGGCAACAGACAT	NM_031189	60
MyHC	F: CGCAAGAATGTTCTCAGGCT<br>R: GCCAGGTTGACATTGGATTG	NM_030679	60
MURF1	R: GCTGGTGGAAAACATCATTGACATC<br>F: GCTGGTGGAAAACATCATTGACATC	NM_001039048	59
Atrogin	R: CTGAATAGCATCCAGATCAGCAGG<br>F: TTGATAAAGTCTTGAGGGGAAAGTG	NM_026346	57

Table S3. Primer sequences and parameters of miR-199a-3p target genes for real-time PCR.

Gene	Primer sequence (5' to 3')	Accession No.	T_m/°C
IGF-1	F: AGGCATTGTGGATGAGTGTT<br>R: TCCTTTGCAGCTTCGTTT	NM_010512	58
PIK3r1	F: CTGAGATTGCTTCGTGAC<br>R: CTTATCCCATGGCTATGA	NM_001077	61
mTOR	F: TCCGAGAGATGAGTCAAGAGG<br>R: CACCTTCCACTCCTATGAGGC	NM_020009	61
RKS6KA6	F: AACGGTTATGCGGGAGC<br>R: AACTGTGCGGGATCTGC	NM_025949	60
GAPDH	F: GGCAAGTTCAACGGCACAG<br>R: CGCCAGTAGACTCCACGACAT	NC_005103	60

Table S4. Primer sequences used for cloning 3' UTR of miR-199a-3p target genes.

Gene	Primer sequence (5' to 3')	Length	Position (NCBI)
IGF-1	F: CCGCTCGAGCTGTGGGTGGGTGAGTAC<br>R: ATTTGCGGCCGCATGCTTTGAATGGGAATG	691	4851-5542
PIK3r1	F: CCGCTCGAGGGTTGACTCTGGGCTTTGC<br>R: ATTTGCGGCCGCCATGGCTATGATCACTCTGGT	681	4046-4727
mTOR	F: CCGCTCGAGAAACCACGTCGTCTCCTC<br>R: ATTTGCGGCCGCAGCATATCCCTCCCTCAC	625	7783-8408
RKS6KA6	F: CCGCTCGAGCTACCTTGAATCCTATGGCG<br>R: ATTTGCGGCCGCGACAAAATACTACACCAAGACTAAAT	660	3682-4342

Underlined symbols are the Restriction Enzyme cutting sites.

© 2013 by the authors; licensee MDPI, Basel, Switzerland. This article is an open access article distributed under the terms and conditions of the Creative Commons Attribution license (http://creativecommons.org/licenses/by/3.0/).
